# YOLO-RDM: A high accuracy and efficient algorithm for magnetic tile surface defect detection with practical applications

**DOI:** 10.1371/journal.pone.0328815

**Published:** 2025-07-18

**Authors:** Wei Niu, Cheng Lv, Enxu Zhang, Zhongbin Wei

**Affiliations:** School of Mechanical Engineering, Xijing College, Xi’an, China; Kafkas University: Kafkas Universitesi, TÜRKIYE

## Abstract

As the core components of permanent magnet motors, the surface defects of magnetic tiles can directly affect the working performance of the motors. There are various types of defects in magnetic tiles, such as chipping and wear. Traditional magnetic tile defect detection mainly relies on manual inspection and faces challenges like low detection accuracy and high cost. Moreover, most defects on magnetic tile surfaces are located on curved surfaces, leading to an uneven distribution of defects and tiny features, which makes accurate defect localization challenging. To solve these problems, this study proposes a novel magnetic tile defect detection algorithm called YOLO-RDM. First, we apply DOConv to the neck network. By using a lightweight convolution method, we replace the traditional convolution in the original network, thereby improving the feature extraction ability of the model and achieving lightweight processing. Second, we design an RPA Block to improve the C2f module. By introducing a parallel attention mechanism, we enhance the feature extraction ability of the algorithm. Finally, we replace the original backbone network of YOLOv8 with the MogaNet network. MogaNet is a module that aggregates contextual information, enhancing the network’s discriminative power, learning efficiency, and ability to capture defect features in images. The experimental results show that the mean average precision (mAP@0.5) of the improved model reaches 95.0%, which is 4.8% higher than that of the original model, and its inference time is less than 5.6 ms. It also has obvious performance advantages compared with other object detection models. In addition, it achieves good recognition results on the NEU metal surface defect dataset. It can be proven that the YOLO-RDM model has strong recognition and generalization abilities and can be used in practical applications of magnetic tile defect detection.

## I. Introduction

Permanent magnet direct current (PMDC) motors have gained widespread application in various fields such as automotive [1–3], aerospace [4,5], and home appliances [6,7] due to their significant advantages including high efficiency, fast response, long lifespan, compact size, and low maintenance costs. As the core power source for these critical industries, PMDC motors’ performance and reliability directly impact the operational efficiency and service life of the entire system. Among these components, magnetic tiles, the core element of PMDC motors, play a crucial role. Magnetic tiles are wafer-shaped permanent magnet materials, and even minor surface defects can significantly affect motor performance. However, due to limitations in manufacturing processes, magnetic tiles are prone to surface defects such as holes, cracks, and wear during production [8]. These defects not only reduce motor operational efficiency but may also lead to motor failures, drastically shortening their service life and causing severe impacts on normal motor operation. As a result, developing an efficient and accurate method for magnetic tile defect detection is of great importance for ensuring optimal PMDC motor performance and minimizing the risk of operational failures.

Traditional methods for magnetic tile defect detection primarily rely on manual visual inspection, which has numerous limitations. First, manual inspection is time-consuming and inefficient, failing to meet the demands of large-scale industrial production. Second, due to the small size of magnetic tiles, their surface defects are often minute and inconspicuous, making it easy for human inspectors to miss or misidentify defects, resulting in unstable detection outcomes. Additionally, manual inspection is highly subjective, with significant variations in detection results among different inspectors, further reducing the reliability and consistency of the process. As such, traditional manual detection methods are no longer sufficient to meet the modern industrial demand for efficient and accurate defect detection.

Recent advancements in computer vision technology have brought revolutionary changes to the field of industrial inspection. Computer vision utilizes automated image processing and analysis to effectively identify and locate defects on object surfaces, significantly reducing labor costs and detection time while minimizing human errors. In the domain of metal surface defect detection, computer vision technology holds immense potential. Object detection, as a key branch of computer vision, aims to identify and locate multiple target categories within images or videos, providing strong technical support for industrial inspection. Currently, mainstream object detection algorithms can be categorized into single-stage and two-stage approaches. Single-stage detection algorithms predict the category and location of objects directly within a single network, offering high detection speeds. Examples include the YOLO series [9], SSD [10], and RetinaNet [11], which have found extensive applications in industrial inspection.

The YOLO series, a representative of single-stage detection algorithms, has evolved through multiple iterations, from YOLOv3 [12] and YOLOv4 [13], followed by subsequent versions such as YOLOv5, YOLOv6 [14], YOLOv7 [15], YOLOv8 [16], YOLOv9 [17], and YOLOv10 [18]. SSD and YOLO-based single-stage algorithms perform object detection using multi-scale feature maps, predicting multiple bounding boxes at each location of every feature map. This enables effective handling of objects of different scales and proportions. These single-stage algorithms excel in industrial inspection due to their fast speed and high efficiency. In contrast, two-stage detection algorithms such as R-CNN [19], Faster R-CNN [20], and Mask R-CNN [21] first generate candidate regions, then classify these regions and perform bounding box regression to generate final detection results. While these algorithms offer high detection accuracy, they are relatively slow. Therefore, in the context of magnetic tile defect detection, which requires a balance between precision and speed, optimizing algorithm performance presents a significant challenge.

In addition, vision detection models based on the hybrid CNN-Transformer architecture have been widely adopted. By leveraging Transformer’s contextual modeling capabilities, these models focus more on target regions and exhibit robust learning abilities. Abhishek et al. proposed a novel hybrid architecture named CorForm for steel structure corrosion inspection. By fusing Transformer and CNN layers at different stages of the encoder, and combining the context-capturing ability of Transformer with the region-aware capability of CNN, they effectively improved the recognition accuracy by 2.7% [22]. Ali et al. achieved the recognition of nine common defects in hot-rolled steel strips by combining Vision Transformer (ViT) and CNN. This method addressed common data challenges through preprocessing techniques. Subsequently, by comparing the proposed model with standalone ViT and CNN, the recognition performance was improved by more than 6% [23]. Chen et al. attempted to embed the Transformer structure into a GAN network for IC metal packaging surface defect detection. By incorporating a cross-scale fusion module, they effectively enhanced the GAN’s processing performance and increased the classification model’s recognition accuracy to 99% [24]. Although Transformer-based vision detection models have been widely used, they suffer from computational complexity and long training times, limiting their expected performance to fixed environments such as laboratories. Further lightweight optimization is still required to achieve practical industrial applications [25].

To address the specific requirements of magnetic tile defect detection, this study proposes an improved defect detection algorithm based on the YOLOv8 detection framework. By optimizing the network structure and incorporating novel modules, the proposed algorithm significantly enhances detection accuracy and efficiency. The main contributions of this research are as follows:

1. Replacement of traditional convolutional layers in the Neck network with Depthwise Over-parameterized Convolutional Layers (DOConv). DOConv introduces additional depthwise convolution operations within standard convolutional layers to create an over-parameterized convolution layer. In the context of magnetic tile defect detection, DOConv improves the precision of defect feature extraction while reducing the number of model parameters and computational requirements.
2. Integration of a newly designed Residual Parallel Attention Mechanism (RPA) into the Neck network to enhance the original c2f module. RPA processes channel and spatial attention in parallel while incorporating residual structures, enabling more efficient attention weight acquisition and improving the model’s perception of critical regions. In magnetic tile defect detection tasks, RPA significantly strengthens the model’s feature extraction capabilities, particularly in complex scenarios where attention allocation to defective areas is more precise.
3. Application of the MogaNet network architecture to the Backbone network, enhancing the network’s discriminative ability and learning efficiency while improving feature fusion capabilities. MogaNet introduces multi-stage feature aggregation modules to effectively encode local perception and contextual information, enhancing the model’s ability to process complex feature interactions. Additionally, MogaNet incorporates an efficient channel aggregation block with adaptive channel redistribution to reduce redundant channel information. In the context of magnetic tile defect detection, MogaNet significantly improves model discriminative ability and learning efficiency, strengthening the model’s capacity for complex defect feature fusion.

## II. Related works

### A. Traditional methods

Traditional approaches in ceramic tile defect detection primarily rely on machine learning techniques, where models are constructed to identify patterns and structures within data for predictive or classification tasks. Huang [26] proposed a defect detection method integrating Wavelet Packet Transform (WPT), Linear Discriminant Analysis (LDA), and Support Vector Machine (SVM). This methodology first extracts normalized image features through WPT, then optimizes SVM parameters using LDA for internal defect identification. Yao [27] implemented self-supervised learning for Printed Circuit Board (PCB) defect detection, employing relative position estimation, spatial adjacency similarity metrics, and K-means clustering for semantic feature learning, complemented by a local image patch completion network for consistent background-local feature integration. Chen [28] pioneered hyperparameter search techniques in defect depth classification, utilizing median filtering for infrared thermal image denoising. The authors introduced two temporal temperature features (transition temperature features and temperature difference features), optimizing KNN, SVM, and Random Forest models through grid and random search. Lee [29] applied explainable machine learning to predict and interpret geometric characteristics and defect types in iron-nickel alloys during metal additive manufacturing. Their framework combines Gaussian Process Regression for alloy height/porosity prediction with SVM-based defect classification, enhanced by SHAP (Shapley additive explanation approach) for feature importance analysis.

### B. Deep learning based defect detection method

With the rapid advancement of deep learning technologies, researchers have increasingly applied these techniques to ceramic tile defect detection. Hou [30] developed RC-YOLOv5s, an enhanced YOLOv5s model incorporating Res-Head and Drop-CA modules to improve cross-layer feature fusion and mitigate model overfocusing on defect targets, thereby reducing missed detection rates. Chen [31] systematically reviewed deep learning’s potential in industrial quality inspection and created a tile defect detection system combining YOLOv5 with MobileNetV3, achieving significant efficiency and accuracy improvements through model fusion.

Wang [32] addressed the limitations of ceramic tile defect detection caused by restricted feature representation and complex backgrounds by modifying YOLOv5 with a Shuffle Attention mechanism. Comparative studies demonstrated the improved Shuffle Attention YOLOv5’s superior detection speed and performance. Wang [33] proposed SSM-R-CNN, a computer vision algorithm integrating SURF-based registration (addressing low contrast and morphological variations) with a SENet-enhanced Mask R-CNN. This approach introduces adaptive weighting through channel-wise feature interaction analysis.

Cui [34] introduced SDDNet, featuring two novel components: the Feature Retention Block (FRB) preserves texture information vulnerable to downsampling via multi-resolution pyramid feature fusion, while the Skip Dense Connection Module (SDCM) facilitates fine-grained detail propagation for improved micro-defect detection. Li [35] developed the Convolutional Retinal Attention Block (CRAB), combining multi-resolution processing with global/local attention aggregation for robust defect classification.

Recent architectural innovations include Lu [36]‘s WSS-YOLO (based on YOLOv8) with WloU loss, Dynamic Snake Convolution (C2f-DSC), and GSConv/VOV-GSCSP modules for adaptive receptive field adjustment. Huang [37] enhanced YOLOv5 through LCA attention mechanisms and a weighted bi-directional feature pyramid, complemented by decoupled head design for precision improvement. Zhao [38] proposed RDD-YOLO with Res2Net backbone and dual-FPN neck architecture for steel defect detection.

Zhang [39] improved industrial gear inspection by integrating CBAMC3 (CBAM-C3 hybrid) attention modules with BiFPN_concat for defect feature fusion, optimized through cosine annealing learning rate scheduling. Their lightweight YOLOv5s variant [40] achieved 5.51% mAP improvement via MobileNetv3 backbone, weighted bi-FPN neck, and Efficient IoU loss. Lv [41] enhanced YOLOv8 for steel plate defects using MobileViTv2 backbone and Cross-Local Connectivity (CLC) modules for improved feature fusion under complex textures. In the field of industrial inspection, the variants and improvement methods of YOLO object detection models are presented in Table 1.

**Table 1:** Variants of YOLO object detection models in the field of industrial inspection.

Author	Base Model	Attention	Backbone	Head
Wenqing, Hou [30]	YOLOv5	Drop-CA		Res-Head
Yuejun, Chen [31]	YOLOv5		MobileNetV3	
Hongdong, Wang [32]	YOLOv5	Shuffle Attention		
Jing, Huang [37]	YOLO v5	LCE attention		BiFPN
Chao, Zhao [38]	YOLO v5		Res2Net	DFPN
Shuwen, Zhang [39]	YOLOv5	CBAMC3		BiFPN_concat
Yinsheng, Zhang [40]	YOLOv5	CBAM	Mobilenetv3	
Zhongliang, Lv [41]	YOLOv8		MobileViTv2	Cross-Local Connection

## III. Materials and methods

### A. YOLOv8

**Fig 1:**
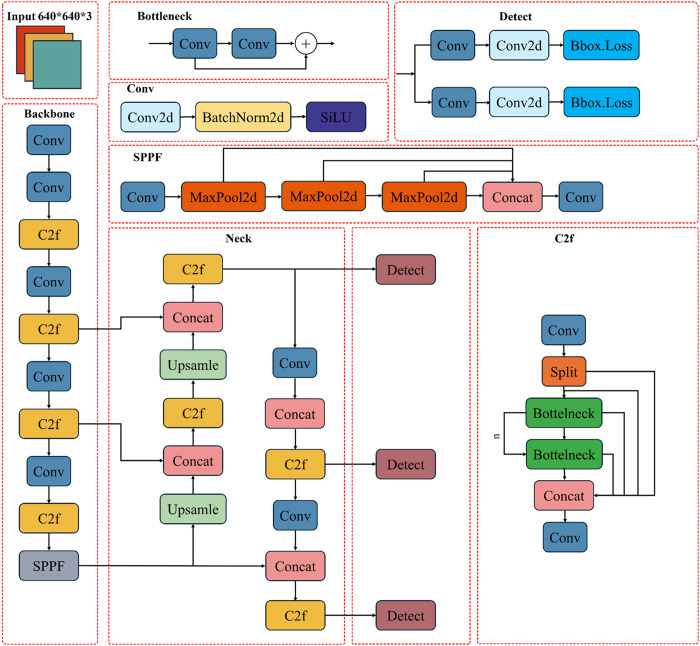
Detailed architecture of the YOLOv8 model.

YOLOv8, as a cutting-edge model in real-time object detection, consists of four critical functional units in its system architecture: input layer, backbone network, neck network, and head network (as shown in Fig 1). The backbone network employs an improved Cross-Stage Partial Network (CSPNet) architecture, which partitions input feature maps into two parallel branches through a feature splitting strategy. The main branch performs successive convolution operations for feature abstraction, while the auxiliary branch preserves original feature information through skip connections, significantly enhancing gradient propagation efficiency.

Specifically, the backbone network comprises C2f modules, standard convolutional layers (Conv), and Spatial Pyramid Pooling Fast (SPPF) modules. The C2f module innovatively integrates the C3 module from YOLOv7 with ELAN design principles and adopts a three-stage processing flow of Split-Bottleneck-Concat. Compared to YOLOv5’s C3 module, this architecture reduces parameter count while achieving richer gradient flow retention. The SPPF module constructs multi-scale feature pyramids through cascaded max-pooling operations, demonstrating superior computational efficiency over traditional SPP modules and substantially improving multi-scale defect feature capture capabilities.

In the feature fusion stage, YOLOv8’s neck network utilizes a bidirectional feature pyramid architecture that organically combines the advantages of Feature Pyramid Network (FPN) and Path Aggregation Network (PAN). The FPN path injects high-level semantic information into low-level feature maps through top-down feature propagation, effectively enhancing recognition capability for small-scale defects. Complementing this, the PAN path fuses low-level spatial details into high-level features via bottom-up feature transmission, significantly improving defect localization accuracy. Notably, this architecture innovatively omits convolutional operations during upsampling stages, reducing computational complexity while maintaining feature fusion quality—a critical advantage for industrial real-time detection scenarios.

Regarding head network design, YOLOv8 adopts a Decoupled Head structure that independently optimizes classification and regression branches through task-specific separation. This structure incorporates Distribution Focal Loss (DFL) to address bounding box discretization issues. Simultaneously, the model employs an Anchor-Free detection paradigm that dynamically generates candidate regions through a Center Prior mechanism, achieving substantial accuracy improvements compared to traditional anchor-based methods. Furthermore, the triple detection head structure optimizes defects of varying scales, effectively resolving scale sensitivity in bounding box regression when combined with the CIoU loss function.

### B. YOLO-RDM

**Fig 2:**
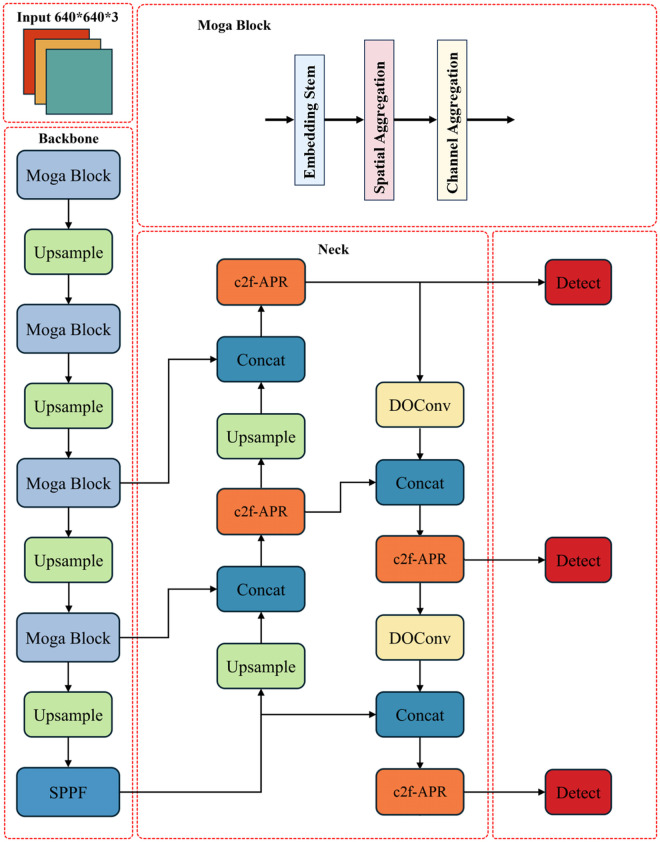
Detailed structure of YOLO-RDM.

To address special challenges in magnetic tile surface defect detection—including low contrast, micro-defects, and complex texture interference—this study proposes an enhanced YOLO-RDM detection model (as shown in Fig 2). The model achieves breakthrough improvements through three heterogeneous modules: RPA Block, DOConv [42], and MogaNet [43]. First, Depthwise Over-parameterized Convolution (DOConv) is introduced into the neck network and attention modules, whose core innovation lies in constructing a hyper-parameterized kernel space to enhance feature representation through multi-branch structures during training, while employing kernel fusion technology for zero-computation augmentation during inference. Second, the RPA Block integrates dual-path mechanisms of channel attention and spatial attention, combined with inverted residual blocks to enhance channel-wise feature map processing and micro-defect focusing capability. Finally, MogaNet replaces the original backbone network, where its gated aggregation mechanism dynamically adjusts multi-scale feature contributions. Coupled with the dual-branch structure of Spatial Mixer (SMixer), this effectively suppresses complex background interference and strengthens network attention to micro-defects. This section details the composition of different structural modules in the YOLO-RDM network.

#### 1) Residual parallel attention mechanism.

**Fig 3:**
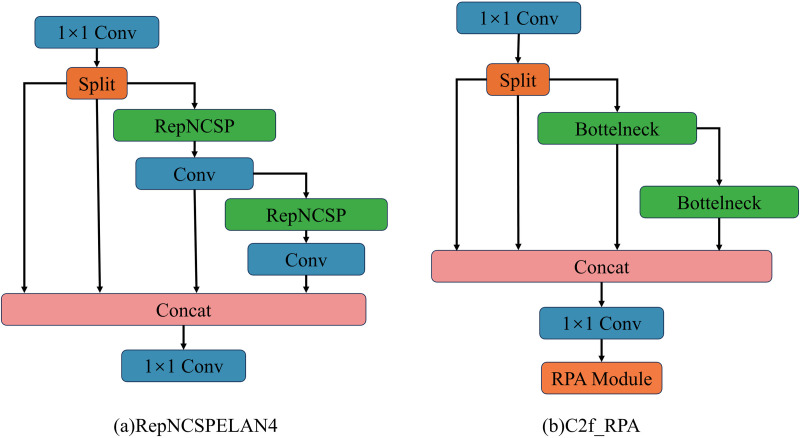
Specific structures of different modules. (a) RepNCSPELAN4 structure, (b) C2f_RPA structure.

This study proposes a Residual Parallel Attention Block (RPA) to enhance the C2f module in YOLOv8n, with its structure illustrated in Fig 3(b). To provide an intuitive comparison between the proposed method and the YOLO series models, Fig 3(a) introduces the RepNCSPELAN4 module from YOLOv9 as a reference. As shown in the figure, the proposed C2f_RPA structure primarily innovates in multi-scale fusion and attention mechanisms. By feeding the multi-scale features output from the Bottleneck into the RPA structure, it leverages parallel channels and attention mechanisms to locate key feature regions in image samples, thereby improving the feature representation capability of the original model.

**Fig 4:**
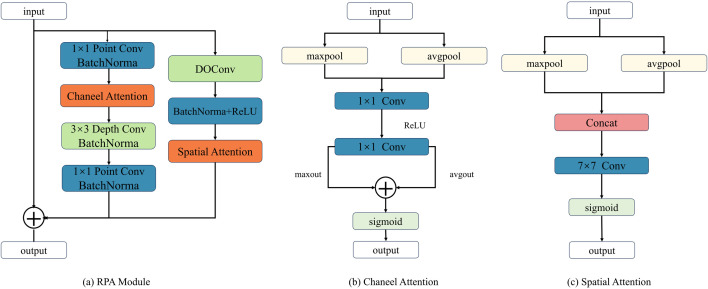
Structure of the RPA Attention Mechanism. (a) RPA attention mechanism structure, (b) channel attention mechanism, (c) spatial attention mechanism.

The core architecture of the RPA module is depicted in Fig 4(a), which adopts a dual-branch parallel processing structure to model attention weights in both channel and spatial dimensions. The left branch, dedicated to channel attention, consists of pointwise convolution, a channel attention mechanism, and depthwise separable convolution. The input feature maps first undergo channel expansion via the first pointwise convolution, followed by the channel attention mechanism to obtain channel weights. Subsequently, the second pointwise convolution restores the original channel count while retaining the core channels through the channel weights. In the channel attention mechanism, the input feature maps are processed by max pooling and adaptive average pooling to extract channel information, which is then passed through a multilayer perceptron (MLP) and a sigmoid function to generate channel attention weights, enabling dynamic weighting of key channels. The computational process is described in Equation 1, and the structure is illustrated in Fig 4(b).

The right branch, focused on spatial attention, is primarily composed of DOConv and a spatial attention mechanism in series. The input feature maps first undergo DOConv to extract spatial information from complex images. In the spatial attention mechanism, spatial semantic information is obtained through average pooling and max pooling, while long-range spatial dependencies are captured using a 7 × 7 large convolutional kernel. The computational process is described in Equation 2, and the structure of the spatial attention mechanism is shown in Fig 4(c). Both branches incorporate BatchNorm normalization layers and ReLU activation functions. The former stabilizes the training process by normalizing feature distributions, while the latter enhances the model’s ability to express complex features through nonlinear transformations. This parallel architecture, through the collaborative optimization of channel and spatial attention, strengthens the model’s ability to model multi-dimensional features without significantly increasing computational costs.


$$Y_{channel}=Sigmoid\left(mlp\left(\max pool\left(X\right)\right)+mlp\left(avgpool\left(X\right)\right)\right)$$ (1)

$$Y_{Spatial}=sigmoid\left(Conv_{7\times 7}\left(Concat\left(\max pool\left(X\right),\;avgpool\left(X\right)\right)\right)\right)$$ (2)

#### 2) Depthwise over-parameterized convolutional layer.

In YOLO models for object detection, convolutional operations are the core component of feature extraction, effectively capturing regional information within samples. In this paper, we enhance the YOLOv8n model by introducing DOConv (Dynamic Overparameterized Convolution), which forms an overparameterized convolutional layer by adding additional depthwise separable convolutions. This method improves the model’s feature extraction capability without introducing additional inference computation. In this study, the convolution is introduced into both the Neck and RPA modules for improvement, as shown in Figs 2 and 4, respectively.

**Fig 5:**
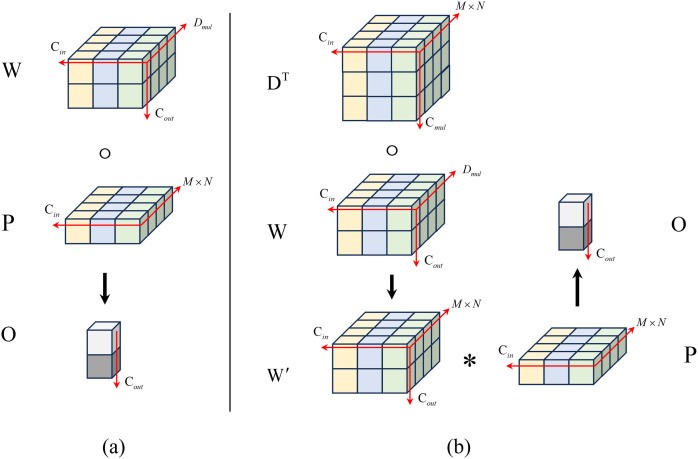
Schematic diagram of DOConv convolution calculation flow. (a) Traditional convolution processing flow, (b) DOConv processing process.


In traditional convolutional operations, the convolution kernel is applied to the windowed regions of the input feature map in a sliding window manner. Assuming the input channel number is $C_{in}$ and the convolution kernel size is, the windowed region acted upon by the convolution kernel can be represented as a 3D tensor, denoted as $P\in R^{M\times N\times C_{in}}$, or equivalently as a 2D tensor, $P\in R^{\left(M\times N\right)\times C_{in}}$. Further assuming the output channel number is $C_{out}$, the convolution kernel is typically represented as a tensor $W\in R^{C_{out}\times \left(M\times N\right)\times C_{in}}$. Using * to denote the convolution operation, the convolution output O=W*P can be expressed as shown in Equation 3, and the convolution process is illustrated in Fig 5(a).

$$O_{C_{out}}=\sum {\mathrm{\backslash nolimits}}_{i}^{\left(M\times N\right)\times C_{in}}W_{C_{out}i}P_{i}$$ (3)

In this paper, to effectively enhance the model’s feature extraction capability, the traditional convolution method is replaced with DOConv. This approach introduces depthwise separable convolution into the convolution process and then applies traditional convolution to the intermediate variables to obtain the output feature map. First, the input feature P is processed with a depthwise convolution using weight $D\in R^{\left(M\times N\right)\times D_{mul}\times C_{in}}$, followed by a traditional convolution using weight $W\in R^{C_{out}\times D_{mul}\times C_{in}}$ (where $D_{mul}\geq M\times N$). The computational process is illustrated in Fig 5(b), and the operation can be expressed as $O=\left(D^{T}\circ W\right)*P$ During the computation, the product of the two weights is first calculated to obtain a new weight $W^{\prime }=D^{T}oW$, which is then used to perform the traditional convolution operation $O=W^{\prime }*P$ on the input feature P to yield the final result. Notably, the receptive field for the input feature remains M×N, and no additional computational operations are introduced due to the extra steps.

Furthermore, in DOConv, both D and W weights are used during training, resulting in more parameters ($\left(M\times N\right)\times D_{mul}\times C_{in}$) compared to traditional convolution. However, during actual inference, the model processes samples using the $W^{\prime }=D^{T}oW$ parameters computed during training, without increasing the computational load during inference. In the context of magnetic tile surface defect recognition, the DOConv-enhanced model theoretically increases computational operations during training but effectively improves feature extraction without adding extra inference operations.

#### 3) MOGANET.

**Fig 6:**
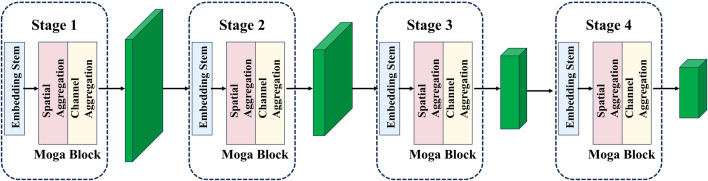
Diagram of the MogaNet model.

**Fig 7:**
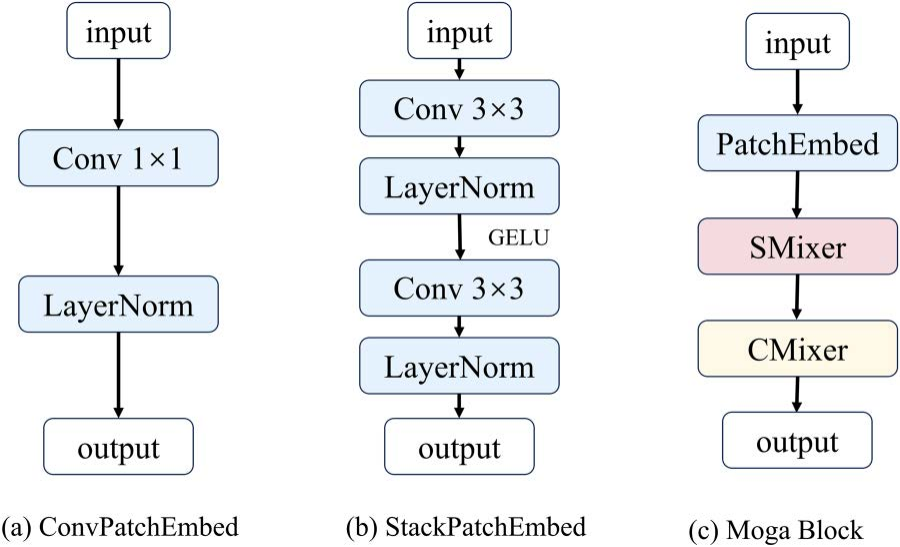
Structure of Moga Block. (a) ConvPatchEmbed structure, (b) StackPatchEmbed structure, (c) Moga Block structure.

In this study, we replaced the backbone network of the YOLOv8 model with the MogaNet network to effectively enhance the model’s capability for defect feature extraction. As a fully convolutional network framework, MogaNet enhances feature extraction for complex targets through multi-level feature fusion mechanisms and dynamic context-aware modules, as illustrated in Fig 6. The framework consists of four feature extraction stages, with resolutions progressively decreasing from H/4 × W/4 to H/32 × W/32, to hierarchically capture multi-scale defect localization information. Each MogaNet Block comprises an Embedding Stem, a Spatial Mixer (SMixer), and a Channel Mixer (CMixer): the Embedding Stem regulates feature resolution and embeds dimensions using learnable downsampling kernels, while the SMixer and CMixer focus on spatial detail enhancement and channel semantic optimization, respectively. Notably, the Embedding Stem in MogaNet has two variants—ConvPatchEmbed (for stages 2–4) and StackPatchEmbed (for stage 1)—optimized for shallow high-frequency information and deep semantic information, respectively, as shown in Figs 7(a) and 7(b).

The MogaNet architecture employs the Moga Block as its core feature extraction unit, as shown in Fig 7. This structure adopts a “spatial-channel dual-path collaboration” strategy: the input feature map undergoes PatchEmbed preprocessing, followed by sequential processing through the SMixer and CMixer for spatial detail modeling and channel relationship modeling, respectively. Finally, multi-level information is fused via residual skip connections, as described in Equation 2. In the Moga Block, the input feature map is first processed by PatchEmbed to obtain the processed feature map and image dimensions, which are then sequentially fed into the SMixer and CMixer and connected using skip connections, as described in Equations 5 and 6.


Z=Stem\nolimits(X) (4)

Y=X+SMixer\nolimits(Norm\nolimits(X)) (5)

Z=Y+CMixer\nolimits(Norm\nolimits(Y)) (6)

**Fig 8:**
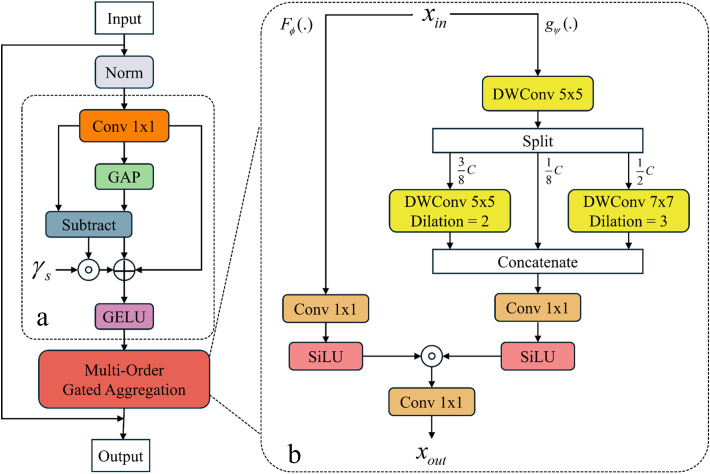
Spatial aggregation structure in the MogaNet network. (a) Adaptive spatial compression structure, (b) Multi – order gated aggregation module structure.


Specifically, the SMixer structure is shown in Fig 8, where Fig 8(a) illustrates the adaptive spatial compression structure and Fig 8(b) depicts the multi-order gated aggregation module. The input feature map is first normalized using LayerNorm to obtain feature map Y. In the adaptive spatial compression structure, convolution operations and adaptive average pooling compress the spatial dimensions of the feature map to 1 × 1, yielding the global average feature Y, as described in Equation 7. Additionally, a learnable detail sensitivity weight factor $\gamma_{s}$ is introduced to dynamically weight local feature differences, enhancing defect features and suppressing background information to produce output data Y or $X_{in}$, as described in Equation 8. Further, the resulting data $X_{in}$ is fed into the multi-order gated aggregation module, where the feature is split into high-frequency component $G_{\psi }\left(\cdot \right)$ and low-frequency component $F_{\phi }\left(\cdot \right)$. These components capture cross-channel feature spatial correlations through multi-scale separable convolutions, and spatial-enhanced feature $X_{out}$ is generated via adaptive weighted fusion, as described in Equations 9–11. This method enhances the model’s robustness to geometric variations in defects.

$$Y=Conv_{1x1}(Norm\left(X\right))$$ (7)

$$Y=X_{in}=GELU(Y+\gamma_{s}⊙(Y-GAP(Y)))$$ (8)

$$X_{g_{\psi }}=DWConv_{5\times 5}\left(Concat\left(\left(DWConv_{5\times 5}\left(X_{in}\right),X_{in},DWConv_{7\times 7}\left(X_{in}\right)\right)\right)\right)$$ (9)

$$X_{out}=\underset{F_{\phi }}{\underbrace{SiLU(Conv_{1x1}(X_{in}))}}⊙\underset{G_{\psi }}{\underbrace{SiLU\left(Conv_{1\times 1}\left(X_{g_{\psi }}\right)\right)}}$$ (10)

$$Y=X+X_{out}=X+Moga(FD(Norm(Y)))$$ (11)

**Fig 9:**
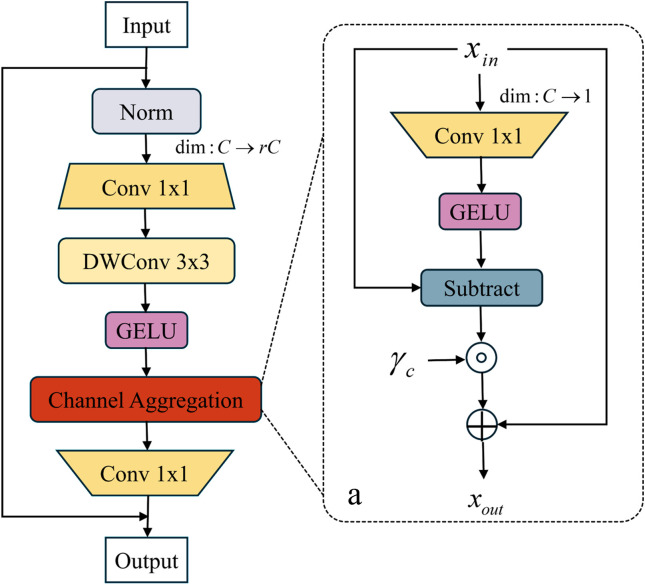
Channel aggregation structure in MogaNet network. (a) Adaptive channel compression structure.


In the channel optimization stage, the CMixer structure processes the input feature, as shown in Fig 9. The feature map undergoes sequential LayerNorm, 1 × 1 convolution mapping, and depthwise separable convolution to generate channel-sensitive feature Y or $X_{in}$, as described in Equation 10. In the adaptive spatial compression structure, the feature map is compressed to a single channel via 1 × 1 convolution, and the semantic deviation between channels is quantified using a global channel offset factor $\gamma_{c}$. $\gamma_{c}$ gated weighting strategy is then applied to enhance the response of key channels, as described in Equation 12. Finally, cross-stage skip connections fuse the original and optimized features, strengthening long-range dependencies between channels while avoiding gradient explosion, as described in Equations 13 and 14. Experimental results demonstrate that this design effectively balances computational efficiency and feature discriminability, providing high-precision, low-redundancy feature representations for complex defect recognition. By introducing this framework into magnetic tile surface defect recognition, the model’s ability to express defect features is significantly enhanced.

$$Y=X_{in}=GELU(DW_{3x3}(Conv_{1x1}(Norm(X))))$$ (12)

$$CA(X)=X_{in}+\gamma_{c}⊙(X_{in}-GELU(Conv_{1\times 1}\left(X_{in})\right))$$ (13)

$$Z=X+X_{out}=X+Conv_{1x1}(CA(Y))$$ (14)

## IV. Experiments

### A. Data set sources and pre-processing

**Fig 10:**
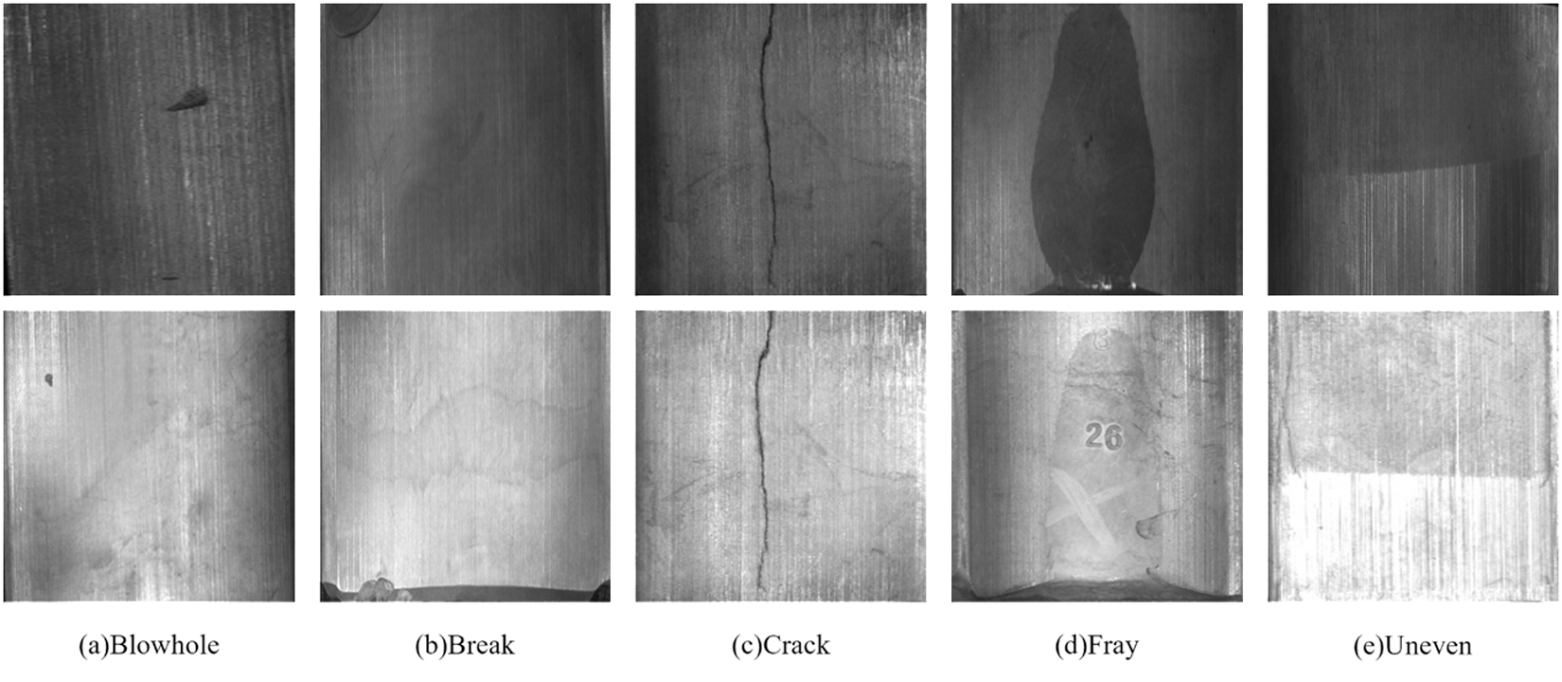
Example diagram of five defects of magnetic tiles.

This study utilizes images from the Magnetic Tile Surface Defects dataset collected on the Kaggle website (https://www.kaggle.com/datasets/alex000kim/magnetic-tile-surface-defects). The dataset contains 1344 grayscale images, categorized into six types: Blowerholes (Blowhole), Breaks (Break), Cracks (Crack), Frazing (Fray), Unevenness (Uneven), and Defect-free (Free). The number of images in each category is 115, 85, 57, 32, 103, and 952 respectively. Notably, the Defect-free category has 952 images, while defective images total 392. The number of defect-free images far exceeds those in other five categories, and each image varies in dimensions. Examples of the five different types of magnetic tile defects are shown in Fig 10.

The dataset suffers from uneven lighting due to site conditions and human operation factors, which reduces the prominence of features in the images. This can negatively impact the accuracy of defect detection during network model training. To address this issue, this paper proposes an image enhancement method based on linear transformations. The method standardizes brightness across different images in the dataset, improving the learning efficiency of neural network models. Its calculation formula is shown as Equation 15.


O(r,c)=a*I(r,c)+b,0≤r<H,0≤c<W (15)

**Fig 11:**
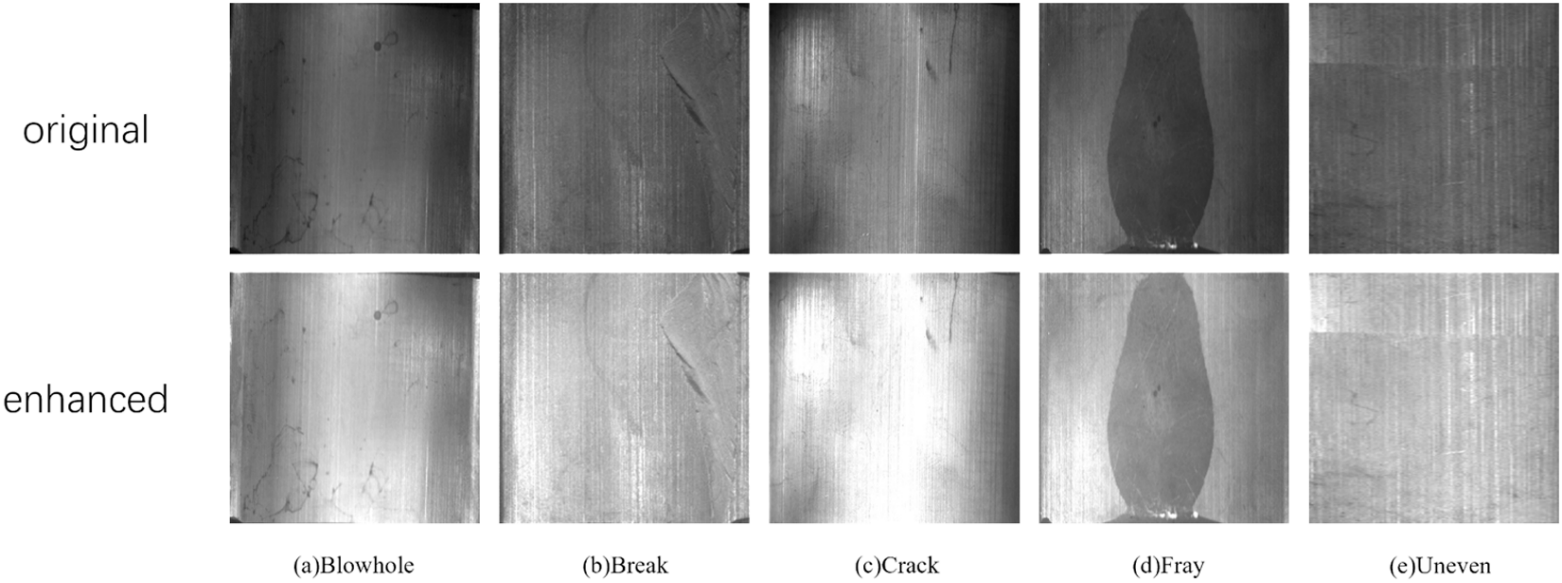
Example of data enhancement.

Where I is the image, W and H denote the width and height of the input image respectively, O is the output image and a is the threshold. As shown in Fig 11. The application of this method significantly improves the contrast of the image and enhances the ability to detect defective targets in magnetic tiles.

It is evident that the sample size of the original dataset is relatively small. To address this issue, data augmentation techniques are commonly used to expand the dataset. Typical data augmentation methods include rotating, scaling, translating, flipping, cropping, and adjusting image contrast. In this study, we applied these methods to augment the dataset, resulting in a total of 1820 images. Subsequently, we annotated the augmented dataset and randomly divided the annotated magnetic tile defect images into training and validation sets at an 8:2 ratio based on their labels. Specifically, the training set contains 1456 images, while the validation set has 364 images.

### B. Experimental parameterization and model training

YOLOv8 is an advanced object detection model that introduces new backbone networks, detection heads, and loss functions compared to previous YOLO versions. It offers multiple models tailored to market demands: YOLOv8n, YOLOv8s, YOLOv8m, YOLOv8l, and YOLOv8x. These models vary in size and performance, with computational efficiency measured in GFLOPs (Table 2). Among them, YOLOv8n is the smallest and most efficient, while YOLOv8x is the largest and most powerful.

**Table 2:** Parameters of each model of YOLOv8 series.

Model	Layers	Params(M)	GFLOPs
YOLOv8n	225	3.15	8.9
YOLOv8s	225	11.12	28.8
YOLOv8m	295	25.9	79.3
YOLOv8l	365	43.69	165.7
YOLOv8x	365	68.23	258.5

Given the requirements of this study, we selected YOLOv8n for its optimal balance between speed and accuracy. The model was trained on NVIDIA hardware with an Intel Core i7-12700 processor and an RTX 3060 GPU, leveraging PyTorch as the primary framework for implementation.

Image size refers to the size of the input image. A larger input image size will affect the speed, accuracy and generalization ability of the algorithm. The YOLOv8 algorithm uses fixed-size images as input to reduce the impact of image size on detection performance. The learning rate affects the update rate of the model weights, which in turn affects the training speed and training stability. The number of iterations (Epoch) represents the number of training rounds, which has a significant impact on the performance of the model. Increasing the number of iterations helps to improve the model performance, but it will increase the model training time. The optimizer is used to continuously adjust the parameters of the model, and the YOLOv8 algorithm can use stochastic gradient descent (SGD) or Adam as the optimizer. In this study, the learning rate is set to 0.01, the number of iterations is 100 rounds, the number of input images per batch is 16, and the optimizer selects SGD(Table 3).

**Table 3:** Training parameters.

Parameter	Configuration
Image size	640 × 640
Learning Rate	0.01
Epoch	100
Batch Size	16

### C. Assessment of indicators

In deep learning target detection networks, models are typically evaluated using Average Precision (AP), Precision, and Recall metrics, where Average Precision measures the accuracy of detecting targets in each category. Mean Accuracy (MAP) represents the average accuracy over multiple categories, and its calculation depends on the IoU threshold. mAP0.5 represents the mAP value at an IoU threshold of 0.5, and mAP0.5-0.95 calculates the mAP values in the range of 0.5–0.95 IoU thresholds before averaging them. This enables a better evaluation of the model’s performance under different IoU thresholds. The mathematical expression for IoU as shown in Equation 16.


$$IoU=\frac{\begin{array}{cc} {}*20cPredicted & region\begin{array}{ccc} {}*20c\;\;\cap & Ground & truth \end{array} \end{array}}{\begin{array}{cc} {}*20cPredicted & region\begin{array}{ccc} {}*20c\;\;\cup & Ground & truth \end{array} \end{array}}$$ (16)

The IoU threshold is used to measure the degree of overlap between the candidate frames generated by the model and the original labeled frames.

Precision is the proportion of positive samples that are correctly predicted by the evaluation model, and Recall is the proportion of all true samples that the evaluation model is able to find out, and they are represented by the Equation 17 and Equation 18.


$$Precision=\frac{TP}{TP+FP}$$ (17)

$$Recall=\frac{TP}{TP+FN}$$ (18)

Where TP denotes true positive, i.e., the predicted IoU is greater than 0.5 and accurately predicted; FP denotes false positive, i.e., the predicted IoU is less than 0.5 or the prediction result is inaccurate; and FN denotes false negative, i.e., the target label is not detected.

The precision-recall (PR) curve shows the relationship between precision and recall of the model at different confidence thresholds, and as the PR curve gets closer to the upper right corner of the axis, it indicates better model performance. The performance of the model is quantitatively measured by calculating the average precision mean (AP), which represents the area under the precision-recall curve. It can be expressed as a mathematical equation (Equation 19).


$$AP=\int_{0}^{1}p(r)dr$$ (19)

The mAP is calculated by adding up multiple AP values and averaging them. The mathematical formula is shown in Equation 20.


$$mAP=\frac{1}{k}\sum_{k=1}^{n}AP_{k}$$ (20)

where k is the number of categories and n is the category of the test object. When k = 1, mAP = AP.

## V. Analysis of experimental results

### A. Graphical analysis of training results

**Fig 12:**
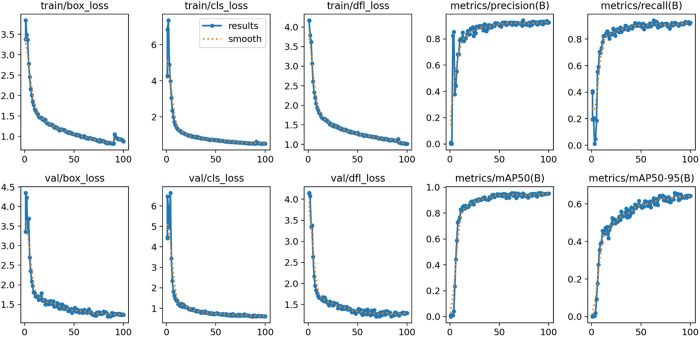
Training results of the proposed YOLO-RDM.

**Fig 13:**
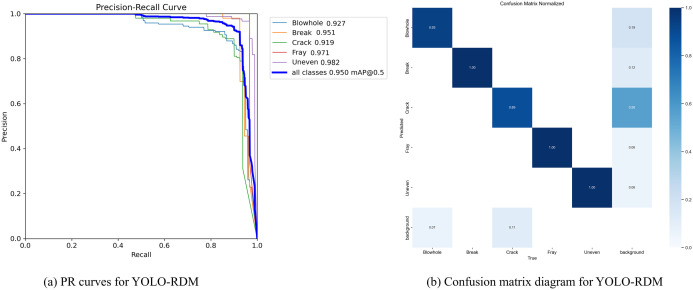
PR curves and confusion matrix test results of YOLO – RDM. (a) PR curves of different defect categories of the model, (b) Confusion matrix test results of the model.

To effectively evaluate the training performance of our proposed YOLO-RDM model, a multi-task joint loss function is employed during model training, including Bounding Box Loss, Classification Loss, and Distribution Focal Loss. The weighted combination of these losses quantifies the multi-dimensional differences between model predictions and ground truth annotations, with the loss convergence process and gradient distribution visualized in Fig 12. As shown in Fig 13(a), the Precision-Recall Curve for the five defect detection tasks exhibits a significant right-skewed characteristic, with an average Area Under the Curve (AUC) of 0.950. The optimal points for Blowhole, Break, Crack, Fray, and Uneven are located at 0.927, 0.951, 0.919, 0.971, and 0.982, respectively, fully validating the model’s ability to balance high sensitivity detection and low false alarm rates. Notably, the mean Average Precision (mAP@0.5) for Uneven achieves an excellent performance of 98.2%, showing a significant 6.3% gap compared to Crack (91.9%). Attribution analysis reveals that the sample size for Crack is only 48.7% of Uneven (214 vs. 439), and the imbalanced data distribution leads to insufficient representation learning. Meanwhile, although Blowhole has a sufficient sample size, its mAP@0.5 is only 92.7% due to its non-uniform spatial distribution characteristics, highlighting the discriminative bottleneck of local features in complex object detection.

**Fig 14:**
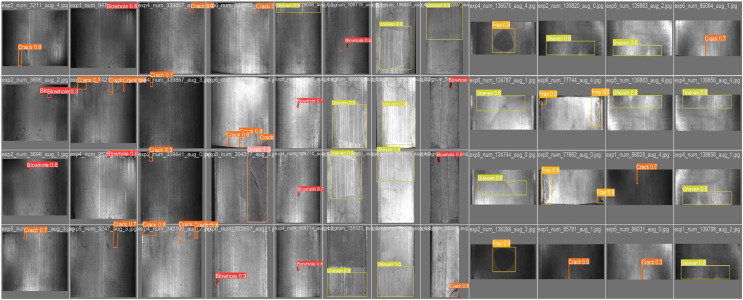
Visualization results.

Fig 13(b) presents the normalized confusion matrix (each row sums to 1), demonstrating the model’s fine-grained classification performance. Ground truth labels for Blowhole, Break, Crack, Fray, and Uneven are correctly classified with high accuracy, with the lowest accuracy for Crack still achieving 89%, confirming the model’s accurate recognition in confusion testing. Additionally, Fig 14 showcases the actual detection results of the best-trained model on dataset samples, accurately identifying defect locations and categories, including spatially uneven defect morphologies.

To verify the training robustness of the model, we conducted systematic validation under different hyperparameter configurations. The test data is shown in Table 4. With Batch Sizes of 8, 16, and 32, the model achieves mAP@0.5 values of 94.2%, 93.2%, and 92.9%, respectively, with an average of 93.43%, indicating greater stability under medium batch sizes (Batch Size = 16). When fixing Batch Size = 16, tests are performed with random seeds 35, 41, and 42, resulting in mAP@0.5 values of 93.2%, 93.6%, and 95.0%, respectively, with a range of only 1.4% and an average of 93.9%. This demonstrates the algorithm’s low sensitivity to parameter initialization and strong robustness.

**Table 4:** Test effects of YOLO – RDM under different conditions.

Methods	Recall/%	mAP@0.5/%	Batch size	Seed
YOLO-RDM	91.58	94.2	8	35
YOLO-RDM	91.5	93.2	16	35
YOLO-RDM	92.0	93.6	16	41
YOLO-RDM	92.9	95.0	16	42
YOLO-RDM	91.38	92.9	32	35

### B. Comparison of different detection models

To validate the superiority of the proposed YOLO-RDM model in object detection, systematic comparative experiments were conducted under identical conditions with mainstream object detection models, including YOLOv5s, YOLOv6s, YOLOv8s, YOLOv9s, YOLOv10s, YOLOv8n, RetinaNet, and SSD. All models were trained under the same conditions using a unified dataset and identical hyperparameter configurations. Evaluation metrics included Recall, mAP@0.5, Params, GFLOPs, and single-frame inference time (Time), with comparative results presented in Table 5.

**Table 5:** Comparative experiments of multiple mainstream target detection models.

Methods	Recall/%	mAP@0.5/%	Params/M	GFLOPs	Times(ms)
YOLOv5s	87.8	90.1	9.11	23.8	6.6
YOLOv6s	88.5	87.5	16.29	44	7.8
YOLOv8s	89.5	92.1	11.12	28.4	7.1
YOLOv9s	88.0	90.5	7.16	26.7	8.7
YOLOv10s	80.3	84.0	8.03	24.5	8.0
RetinaNet	70.4	78.9	32.4	83.1	30
SSD	62.9	75.5	14.43	15.76	30
YOLOV8n	88.9	90.8	2.7	8.6	4.2
Ours	92.9	95.0	4.29	16.6	5.6

Experimental results indicate that SSD and RetinaNet models, based on the ResNet50 framework, achieved a maximum mAP@0.5 of less than 80% under transfer learning strategies. Additionally, as shown in Table 5, these two models exhibited significantly higher parameter counts (32.4M/14.3M) and computational costs (83.1/15.76 GFLOPs) compared to other models, with inference times reaching 30 ms. In contrast, YOLO-series models such as YOLOv5s and YOLOv6s, along with the proposed YOLO-RDM, demonstrated superior recognition performance. Specifically, the proposed YOLO-RDM achieved a mAP@0.5 of 95%, representing a 2.9% improvement over the suboptimal YOLOv8s model, while maintaining real-time inference capability at 5.6 ms.

**Fig 15:**
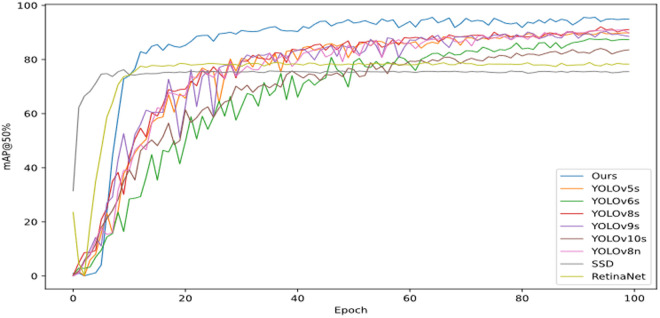
Comparison of average accuracy curves of different models.

Notably, YOLO-RDM demonstrates a reasonable increase in model complexity. Its parameter count (4.29M) and computational cost (16.6 GFLOPs) are 1.53× and 1.93 × higher than those of YOLOv8n, respectively. However, these values remain significantly lower than other comparative models, such as YOLOv5s (9.11M, 23 GFLOPs). As revealed by the training curves in Fig 15, YOLO-RDM exhibits more stable convergence characteristics during training, with substantially reduced fluctuations in its mAP@0.5 curve compared to YOLOv5s. Furthermore, it achieves detection accuracy exceeding 90% by the mid-training phase.

**Fig 16:**
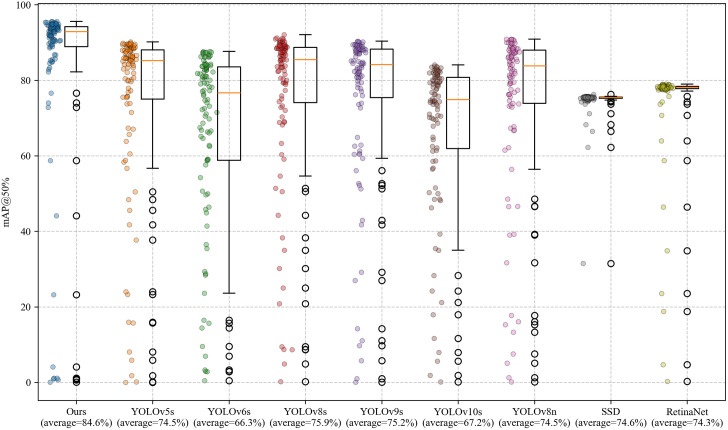
Distribution box plots of different models in the comparative experiment.

To further validate the statistical significance of the model’s performance advantage, this study employs the Wilcoxon signed-rank test instead of the traditional t-test for pairwise comparisons. This non-parametric test method does not require the data to satisfy a normal distribution assumption and evaluates the median difference of paired samples to determine statistical significance, making it particularly suitable for small sample sizes (n < 30) and skewed distribution scenarios [44]. To avoid inflation of the Family-Wise Error Rate (FWER) caused by multiple comparisons, the Bonferroni correction is applied to strictly adjust the significance threshold from α = 0.05 to α = 0.00625 (0.05/8 comparisons), significantly enhancing the robustness of statistical inference. Additionally, Cohen’s d effect size is calculated to quantify the practical efficacy of model improvements, with the following criteria: |d| ≥ 0.8 (large effect), 0.5 ≤ |d| 0.8 (medium effect), and |d| < 0.5 (small effect), providing an interpretable quantitative basis for performance improvements (Table 6). The boxplot of multi-model mAP@0.5 distributions in Fig 16 further supports this conclusion: the median line of our model (orange box) at 84.6% is significantly higher than other models (78.2%−82.4%), indicating that YOLO-RDM achieves both higher detection accuracy and better training stability. Additionally, although SSD and RetinaNet can achieve effective dense distributions, their distribution intervals remain below the lower bound of our model, probabilistically validating YOLO-RDM’s advantage in precision boundary improvement. Through hypothesis testing, effect size quantification, and distribution visualization, this study systematically proves the effectiveness of YOLO-RDM in magnetic tile surface defect detection.

**Table 6:** Statistical effect values of different models.

Methods	YOLOv5s	YOLOv6s	YOLOv8s	YOLOv9s	YOLOv10s	RetinaNet	SSD	YOLOV8n
P Value	4.8 × 10^–^¹⁶	7.1 × 10^–^¹⁹	1.2 × 10^–^¹⁴	3.1 × 10^–^¹⁵	2.5 × 10^–^¹⁸	5.6 × 10^–^¹¹	3.8 × 10^–^⁹	2.0 × 10^–^¹⁵
Cohen’s d	0.431	0.775	0.385	0.418	0.786	0.594	0.534	0.437

### C. Ablation experiment

To validate the effectiveness of different algorithmic module improvements, this study performs a multi-dimensional evaluation of the model based on ablation experiments. In the experiments, we use YOLOv8n as the baseline model and gradually integrate SAConv, DOConv, Ghost backbone network, MobileNetv4 backbone network, Parallel Residual Aggregation Attention Mechanism (RPA), and the fully convolutional MogaNet backbone network. By setting the initial hyperparameters (Batch Size = 16, random seed = 42) for training, we analyze the model’s impact in terms of recall, mAP@0.5, parameter count (Params), and floating-point operations (GFLOPs). The experimental results are shown in Table 7.

**Table 7:** Ablation experiments performed on each improved module.

Methods	Batch Size	Recall/%	mAP@0.5/(%)	Params/M	GFLOPs
YOLOV8n	16	88.9	90.8	2.7	8.7
YOLOV8n+SAConv	16	83.6	84.0	3.58	6.9
YOLOv8n+GhostNet	16	83.0	85.1	1.71	5.0
YOLOv8n+Mobilenetv4	16	84.4	86.5	5.7	22.5
YOLOv8n+DOConv	16	89.3	91.2	2.52	4.1
YOLOv8n+DOConv + RPA	16	90.4	92.7	2.92	4.4
YOLOv8n+DOConv + RPA+MogaNet	16	92.9	95.0	4.29	16.6

First, we introduce DOConv and SAConv into the baseline model YOLOv8n for comparative testing. After integrating DOConv, mAP@0.5 increases from 90.8% to 91.2%, while computational cost decreases by 4.7% (GFLOPs reduced from 12.3 to 11.7), demonstrating the significant advantages of dynamic convolution in small object feature extraction and computational efficiency optimization. However, after introducing SAConv, the model performance significantly declines (mAP@0.5 drops from 90.8% to 84.0%), and the parameter count increases to 3.58M. Analysis indicates that the switchable atrous convolution in SAConv introduces noise interference and increases computational load in the magnetic tile surface defect detection scenario, leading to reduced feature discriminability.

Next, we introduce GhostNet and MobileNetv4 as backbone networks for testing. GhostNet replaces the original C2f structure with the Ghost Module, achieving model lightweighting (parameter count decreases from 3.2M to 2.8M, GFLOPs decrease from 12.3G to 10.1G). However, mAP@0.5 significantly drops from 90.8% to 85.1%, indicating that GhostNet fails to effectively extract features of magnetic tile surface defects, resulting in a notable negative impact. Similarly, MobileNetv4, which uses Extra Depthwise Inverted Bottleneck as the main feature extraction structure, fails to achieve the expected results. The model performance mAP@0.5 drops from 90.8% to 86.5%, and computational cost GFLOPs significantly increase from 12.3G to 22.5G, indicating insufficient feature representation capability.

Finally, in the joint optimization experiment of attention mechanisms and backbone networks, we introduce the RPA attention mechanism into the YOLOv8n (DOConv version), achieving a significant improvement of 1.9 percentage points in mAP@0.5 (from 90.8% to 92.7%) and a 35.0% reduction in computational cost (GFLOPs decrease from 12.3G to 8.0G). This validates the dual advantages of the attention mechanism in feature selection and computational efficiency optimization. When the backbone network is replaced with MogaNet, the model performance reaches its optimal mAP@0.5 of 95.0%, an improvement of 4.2 percentage points over the baseline model. Moreover, the computational cost (GFLOPs = 16.6G) and parameter count (Params = 4.29M) are both lower than the MobileNetv4 version, fully demonstrating the effectiveness of MogaNet in multi-scale feature fusion and context modeling for magnetic tile surface defect recognition, enabling effective feature representation.

### D Comparative experiments on different datasets

**Fig 17:**
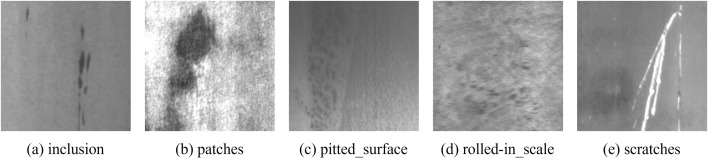
Image samples of NEU metal surface defect dataset.

To comprehensively evaluate the generalization capability of YOLO-RDM, training and testing were performed on the NEU metal surface defect dataset. Identical training models and parameters from the comparative experiments (including YOLOv5s, YOLOv6s, YOLOv8s, RetinaNet, and SSD) were used, as detailed in Table 8. The experiment focused on five defect types: inclusion, patches, pitted_surface, rolled-in_scale, and scratches, with dataset samples illustrated in Fig 17 (available at http://faculty.neu.edu.cn/songkechen/zh_CN/zdylm/263270/list/index.htm).

**Table 8:** Uses different models to train NEU dataset results.

Methods	Recall/%	mAP@0.5/%	Params/M	GFLOPs	Times(ms)
YOLOv5s	75.2	81.7	9.11	23.8	6.6
YOLOv6s	74.2	80.0	16.29	44	8.2
YOLOv8s	77.5	84.1	11.12	28.4	7.6
YOLOv9s	77.2	81.7	7.16	26.7	8.5
YOLOv10s	72.6	77.8	8.03	24.5	8.0
RetinaNet	58.9	75.3	32.4	83.1	31
SSD	54.3	74.1	14.43	15.76	28
YOLOV8n	83.3	86.6	2.7	8.6	4.1
Ours	87.2	89.8	4.29	16.6	6.3

As shown in Table 8, the proposed YOLO-RDM achieves the highest mAP@50 of 89.8%, significantly outperforming traditional detection models (+15.7% vs. SSD) and the baseline YOLOv8n model (+3.2% vs. YOLOv8n). Although its inference time increases by 2.2 ms to 6.3 ms, it remains suitable for rapid detection requirements.

**Fig 18:**
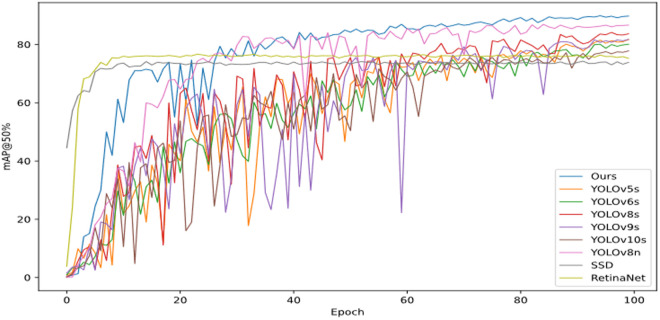
The NEU dataset is used to train the accuracy curves of different models.

The mAP@0.5 curves in Fig 18 reveal distinct convergence patterns: SSD and RetinaNet exhibit rapid early accuracy improvements followed by plateaus, while YOLOv5s, YOLOv6s, and YOLO-RDM show continuous accuracy gains with training progression. Notably, YOLO-RDM demonstrates the smallest fluctuation amplitude, with accuracy never dropping below 40%. In contrast, YOLOv5s and YOLOv9s exhibit the largest fluctuations, with minimum mAP@0.5 values below 20%.

Results from Table 7 and Fig 18 confirm that YOLO-RDM achieves more effective recognition (mAP@0.5: 89.8%), the highest among all compared models, while demonstrating generalization capability across diverse metal surface defects.

## VI. Discussion

In this study, we propose YOLO-RDM, an efficient defect detection model for magnetic tiles, designed to enhance detection accuracy. First, we introduce DOConv to replace traditional convolutions, achieving lightweight processing while improving the model’s feature map processing capability, thereby accelerating convergence speed and reducing model size. Additionally, we propose a novel RPA Block to replace the C2f module in YOLOv8n, enhancing the model’s ability to capture diverse global features and boosting recognition performance. Finally, we adopt the MogaNet backbone to replace the original network in YOLOv8n, leveraging its fully convolutional architecture and high-order multi-gated mechanisms to effectively extract advanced semantic features for precise defect localization.

To validate the recognition capability of YOLO-RDM, comprehensive experiments were conducted. First, comparative tests were performed against mainstream object detection models, including YOLOv3s, YOLOv5s, YOLOv6s, YOLOv8s, RetinaNet, SSD, and the unmodified YOLOv8n. Results demonstrate that YOLO-RDM significantly outperforms other models, with a maximum performance gap of approximately 19.5%, while maintaining minimal inference time changes compared to the original model. Ablation studies further verify the contributions of DOConv, RPA Block, and MogaNet. Among these, introducing MogaNet increases model parameters and computational requirements but elevates baseline accuracy by approximately 5%. Finally, generalization experiments on the NEU metal surface defect dataset confirm that YOLO-RDM achieves superior accuracy over other models, with smaller training curve fluctuations, proving its robustness and adaptability to diverse metal defect detection tasks.

## VII. Conclusion

This study proposes YOLO-RDM, a magnetic tile defect detection model based on YOLOv8n. By integrating Depthwise Overlapping Convolution (DOConv), Region-Aware Attention Block (RPA Block), and MogaNet, the model addresses issues such as uneven distribution of surface defects and microscopic features in magnetic tiles. It leverages a parallel attention mechanism and a fully convolutional backbone network to localize complex feature regions. Extensive experiments validate its robust defect recognition capability, achieving a mean Average Precision (mAP@0.5) of 95.0% at an Intersection over Union (IoU) threshold of 0.5, with an inference time of only a few milliseconds, enabling real-time defect monitoring. However, the model still has certain limitations, including fluctuations in the mAP@0.5 curve during training, slow early convergence, and a relatively large model size. Additionally, the limited dataset used for training and the inherent data imbalance in defect samples pose challenges for model training, experimentation, and research. To address these issues, we will further focus on small-sample processing, model lightweighting, and practical deployment experiments, aiming to conduct in-depth research on real-time defect detection in complex industrial environments.

## Supporting information

S1 Data (ZIP)
